# Effect of inoculated azotobacteria and *Phanerochaete chrysosporium* on the composting of olive pomace: Microbial community dynamics and phenols evolution

**DOI:** 10.1038/s41598-019-53313-z

**Published:** 2019-11-18

**Authors:** Vesna Milanović, Andrea Osimani, Federica Cardinali, Manuela Taccari, Cristiana Garofalo, Francesca Clementi, Selim Ashoor, Massimo Mozzon, Roberta Foligni, Laura Canonico, Maurizio Ciani, Lucia Aquilanti

**Affiliations:** 1 Dipartimento di Scienze Agrarie, Alimentari ed Ambientali, Università Politecnica delle Marche, 60131 Ancona, Italy; 2 Dipartimento di Scienze della Vita e dell’Ambiente, Università Politecnica delle Marche, Via Brecce Bianche, 60131 Ancona, Italy; 3 Department of Agricultural Microbiology, Faculty of Agriculture, Ain Shams University, Cairo, Egypt

**Keywords:** Microbial ecology, Biodiversity

## Abstract

The effect of inoculated azotobacteria and basidiomycetes white-rot fungi on the population dynamics of bacteria and eumycetes during the co-composting of olive mill pomace and wheat straw was evaluated by Polymerase Chain Reaction-Denaturing Gradient Gel Electrophoresis (PCR-DGGE) analysis combined with sequencing of rRNA gene amplicons from selected DGGE bands. The evolution of pH, temperature, phytotoxicity and water-soluble phenol content during co-composting was also monitored. In general, a similar evolution of microbial biodiversity was seen in both the inoculated and uninoculated (control) piles, which was in keeping with a similar evolution of phytotoxicity and water-soluble phenol content. Overall, under the conditions applied, data suggest a marginal influence of the inoculated starters on the physical, chemical and microbiological properties of compost piles, with the resident microbiota playing a major role.

## Introduction

Olive pomace is an olive mill solid waste that is produced during the discontinuous (press-based) olive oil extraction process^1^ and that consists mainly of water, olive seed and pulp. Olive pomace is potentially harmful for the environment due to numerous intrinsic properties, including its acidic pH, relatively high content of salts and organic compounds, phytotoxicity, antimicrobial activity, and phenolic and lipid components^2^. The organic matter within this agro-industrial waste consists mainly of nitrogenous compounds, organic acids, sugars, tannins, pectins, carotenoids, oil residues, and phenolic substances^2^. Over the last decade, the composting of olive pomace has been increasingly attracting the attention of the scientific community due to the beneficial effects of the resulting compost on horticultural and short-term crops^3^. Composting substantially consists in the aerobic stabilization of organic matter^4^. During composting, the endogenous microbiota colonizing the solid matrix being composted transforms the organic matter into CO_2_, biomass, heat and humic compounds^5^.

To date, several microorganisms, including azotobacteria and ligninolytic microfungi, have been assayed, either alone or in consortia, as accelerating agents for the composting of olive mill byproducts. Azotobacteria are free-living nitrogen-fixing bacteria capable of growing^6^ and even fixing atmospheric nitrogen in olive mill wastes^7,8^. Select strains of azotobacteria have been found to remove phytotoxic compounds^9^ and produce both fertility-promoting metabolites^10^ and exopolysaccharides^11^ during aerobic treatment of olive mill wastewater.

Concerning ligninolytic microfungi, *Phanerochaete chrysosporium* (anamorph, *Sporotrichum pulverulentum*) is a white-rot fungus belonging the Basidiomycotina subdivision, which has been recognized as comprising some of the most effective degraders of lignin^5^. So far, *P. chrysosporium* has been extensively exploited in composting trials with numerous agricultural wastes, including olive-mill waste water^12^ and olive pomace^13,14^.

By contrast, only scarce research is available regarding microbial community dynamics and polyphenol evolution during composting of olive mill pomace, and to the best of the authors’ knowledge, only two studies have examined the effect of microbial accelerating agents on the composting of this agro-industrial waste^15,16^. Though both studies investigated the physical and chemical traits of the compost piles, neither identified the bacterial and fungal species that predominated during the composting process.

Given this knowledge gap, this study aimed to investigate the microbial population dynamics and the evolution of polyphenols during the co-composting of olive pomace and wheat straw inoculated with azotobacteria and ligninolytic microfungi, in comparison with uninoculated control compost. To this end, culture-dependent (viable cell counting) and culture-independent (PCR-DGGE) analyses were carried out to monitor the bacterial and eumycete diversity in piles during the composting process; in parallel, physical (temperature, moisture) and chemical (pH, total polyphenols content) properties of the piles were also monitored.

## Materials and Methods

### Composting procedure

Wet olive pomace (WOP) derived from olive oil production during the 2015–2016 season was provided by the “Azienda del Carmine” oil mill (Agugliano, AN, Italy). The WOP was produced by the two-phase system of oil extraction from olives of the “Leccino” cultivar. The composting of WOP was carried out in piles placed in open, plastic 65-L containers to avoid the loss of percolate. The composting trial was carried out in a greenhouse. Four composting piles were prepared, labeled as 1A, 1B, 2A and 2B. For each pile, 20 kg of WOP, 15 kg of wheat straw and 5 kg of wood chips were mixed. At the end of the thermal phase (day 25 of composting) and again after 94 days of composting, experimental piles 1A and 1B were inoculated with a suspension of three azotobacteria strains (see paragraph “Reference strains” for more details). The bacterial inoculants were selected based on their ability to abate water-soluble phenols in olive mill wastewater, as previously assessed by Aquilanti *et al*.^17^. At the beginning of the maturation phase (44^th^ day of composting), piles 1A and 1B were also inoculated with a strain of *P. chrysosporium* (see paragraph “Reference strains” for more details) that was previously assayed in co-composting trials of agricultural wastes mixed with olive mill wastewater^13^. Piles were turned manually three times a week to provide aeration and improve pile homogeneity throughout the composting trial. The average moisture content of the initial mixtures was kept at 48 ± 5% by irrigating with water when necessary.

Samples of approximately 0.3 kg were randomly collected from each pile at three different depths for physical, chemical, and microbiological (culture-dependent and culture-independent) analyses. Samples were collected at regular intervals during the composting process and were vigorously mixed before analysis.

### Reference cultures

Three bacterial reference strains (*Azotobacter chroococcum* 208, *A. chroococcum* 220 and *A. chroococcum* 225) and one fungal strain (*P. chrysosporium* DSMZ 6909) were used to inoculate experimental piles 1A and 1B and to prepare the two DGGE ladders. The three bacterial strains had previously been isolated from soils that were regularly treated with olive mill wastewater; they were molecularly identified as *A. chroococcum* and were assayed for their ability to abate water-soluble polyphenols in sterile CaO-treated olive mill wastewater^17^. The fungal strain was purchased from the Deutsche Sammlung von Microorganismen und Zelkulturen (DSMZ) (Braunschweig, Germany) and reconstituted according to DSMZ recommendations.

### Microbial inoculation

The three *A. chroococcum* strains (208, 220, 225) were cultured separately in tryptic soy broth (TSB) for biomass production. The cultures were incubated on a rotary shaker at 150 rpm. After 48 h incubation at 30 °C, the biomass was harvested by centrifugation, the supernatant was discarded, and the pellet was resuspended in sterile physiological solution 0.85% (w/v) of NaCl. Concentration of the bacterial suspensions was determined using a Thoma Zeiss counting chamber, whereas the cell viability was checked by the plate count method using tryptic soy agar (TSA). Equal parts (150 mL) of the three bacterial suspensions were mixed and used to inoculate the composting piles by spraying with a dispenser. The bacterial suspension of the three *A. chroococcum* strains was inoculated twice during composting, specifically, on the 25^th^ and the 94^th^ day of composting, to achieve a final concentration of 6 and 8 Log CFU g^−1^ compost at the two respective timepoints. The inoculation of *P. chrysosporium* spores was performed on the 44^th^ day of composting to achieve a final concentration of 6 Log CFU g^−1^ compost. The inoculum of fungal spores was prepared as previously described by Taccari *et al*.^13^.

### Physical and chemical analyses

The temperature of each compost pile was measured daily at three depths (upper, middle and bottom part of the pile) using a temperature probe (Escort Junior, ESCORT DATA LOGGING SYSTEM LTD, Auckland, Australia), and the mean value of three measurements was calculated. The ambient air temperature was also measured using a Wet and Dry Bulb Hygrometer (G.H. Zeal, London, UK). The moisture content, pH and concentration of water-soluble phenols in the WOP and compost material collected during composting were determined following the procedures described by Taccari *et al*.^13^. All measurements of moisture, pH, and water-soluble phenols were carried out in duplicate. The results were expressed as mean values ± SDs.

### Phytotoxicity test

WOP and aliquots of the inoculated and control piles were assayed for their phytotoxicity during composting. The phytotoxicity, expressed as germination index (G.I.), was estimated by comparing the root development of cress (*Lepidium sativum*) seeds in contact with compost extracts to that of seeds in contact with deionized water (control) after incubating for 72 h at 25 °C. The test was performed in triplicate following the procedure previously described by Zucconi *et al*.^18^ with some modifications, as reported by Mari *et al*.^19^.

### Microbiological analyses

Total mesophilic and thermophilic aerobes and eumycetes were enumerated on WOP and during composting by viable cell counting. Samples of approximately 0.3 kg were randomly collected at different depths of the four piles at regular intervals during composting and were vigorously mixed. Ten grams (wet weight) of each sample were homogenized in 90 mL sterile peptone water (0.1% peptone) at 260 rpm for 3 min using a Stomacher apparatus 400 Circulator (International PBI, Milan, Italy). Serial dilutions of the suspensions were plated on Plate Count Agar for enumeration of mesophilic (incubated for 24–48 h at 30 °C) and thermophilic (incubated for 24–48 h at 50 °C) bacteria. Rose-Bengal chloramphenicol agar was used to enumerate fungi after incubating for 48–72 h at 25 °C. All samples were analyzed in duplicate, and the mean values were calculated; the results are expressed as Log CFU g^−1^.

### DNA extraction

DNA from the bacterial and fungal reference strains was extracted as described by Garofalo *et al*.^20^ and Makimura *et al*.^21^, respectively. An E.Z.N.A. soil DNA kit (Omega biotek, Norcross, GA, USA) was used to extract microbial DNA directly from the samples collected at regular intervals during composting. A 1-mL aliquot of sample homogenate that had been diluted 10^−1^ for viable-cell counting was centrifuged to produce a pellet that was then processed according to the kit manufacturer’s instructions. The quantity and purity of the extracted DNA were assessed by absorbance at 260, 280 and 234 nm using a UV-Vis Shimadzu UV-1800 spectrophotometer (Shimadzu Corporation, Kyoto, Japan).

### PCR-DGGE analyses

The DNA extracted directly from the composting samples were amplified by PCR using two primer sets, namely, 338f-518r for bacteria^22^ and NL1-LS2 for fungi^23^. A GC clamp was added to the 5’ end of the forward primers, 338 f^24^ and NL1^25^. For all amplification reactions, 100 ng of DNA was amplified in a total reaction volume of 50 µL containing 1.25 U of Taq DNA polymerase (SibEnzyme, Novosibirsk, Russia), 1X reaction buffer (pH 8.5; 60 mM Tris-HCl, 25 mM KCl, 1.5 mM MgCl_2_, 2-mercaptoethanol, 0.1% Triton X-100), 0.2 mM dNTPs, and each primer at 0.2 µM. The PCRs were performed in a My Cycler thermal cycler (Bio-Rad, Hercules, CA, USA) using the programs previously described by Osimani *et al*.^26^. For each PCR, 5 µL of product was analyzed by electrophoresis in 1.5% agarose gels in 0.5X TBE containing 0.5 mg mL^−1^ ethidium bromide at 6 V cm^−1^ for 1 h, using the HyperLadder 100 bp (Bioline, London, UK) as a molecular weight standard. Gels were visualized under UV light and photographed using the Complete Photo XT101 system (Explera, Jesi, Italy).

DGGE analyses of the bacterial and fungal communities were carried out using a DGGE DCode^TM^ system (Bio-Rad), as previously described by Taccari *et al*.^27^. The ladder for the analysis of the bacterial community was prepared by mixing PCR products obtained from the three *A. chroococcum* reference strains. The NL1_GC_-LS2 amplicon of *P. chrysosporium* was used as a ladder in each DGGE gel for the analysis of the fungal community. After electrophoresis, the gels were stained for 20 min in 1X TAE buffer containing 1X SYBR Green I Nucleic Acid Gel Stain (Lonza, Walkersville, MD, USA) and then visualized under UV light and photographed using the Complete Photo XT101 system (Explera).

Select DGGE bands were excised from the gels using sterile cutting tips. DNA from each band was eluted in 50 µL deionized water; 5 µL of eluted DNA was reamplified in a PCR reaction under the same conditions as described above but using forward primers 338 f and NL1 without the GC clamp. The obtained amplicons were sent to Genewiz (Hope End, Takeley, UK) for purification and sequencing. The analysis of the obtained sequences was performed as previously described by Garofalo *et al*.^28^.

### Statistical analysis

Analysis of variance (ANOVA) was applied to assess differences in pH, water-soluble phenol content and phytotoxicity. The means were analyzed using the STATISTICA 7 software (freely available on the internet). Significant differences were determined by the Duncan multiple range test, with P values < 0.05 considered to be significant.

## Results and Discussion

### Physical and chemical analysis of wet olive pomace

Wet olive pomace (WOP) used for the composting showed the following values for the physical and chemical traits considered: pH 5.05 ± 0.02, concentration of water-soluble phenols (expressed as mg Kg^−1^ dry weight) 13’244.12 ± 45.75 and G.I. (%) 19.66 ± 4.83. Concerning the first two parameters, data collected in the present study were within the range reported by other authors for semisolid olive mill wastes produced with two-phase olive oil extraction systems^29^. The water-soluble phenol content of olive mill wastes is known to be greatly dependent on several variables, including the geographical area and conditions, soil composition, ripeness of the fruits, olive cultivar, storage conditions prior to processing, and olive oil extraction system applied^30^.

**Figure 1:**
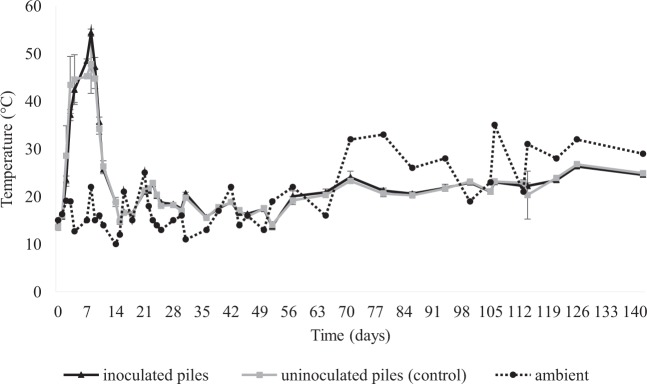
Temperature profile during composting. Temperatures are the average recorded at three different depths in the piles. Inoculated piles (1A and 1B) underwent inoculation with azotobacteria and spores of *Phanerochaete chrysosporium*, whereas uninoculated piles (2A and 2B) were used as controls. Error bars indicate ± SD of two independent experiments.

The temperature profiles of compost piles and the ambient air during composting are shown in Fig. 1. A typical succession of the mesophilic, thermophilic and maturation phases characterizing the static pile process was seen. Because of a rapid warm-up of the piles, the temperatures measured at the middle of the piles reached 40–45 °C in 3–4 days. In all piles except 2A, the temperature increased to 55 °C after 8 days of composting; thereafter, a rapid drop in temperature was seen, and temperatures near 15–20 °C were reached after 14 days. These latter temperatures were roughly maintained until the end of the monitoring period. As expected, at least in the third phase, the internal temperature of the four piles was affected by the ambient air temperature.

**Figure 2:**
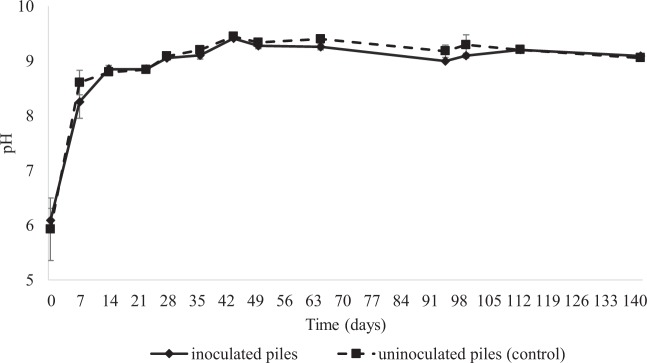
pH evolution of inoculated (1A and 1B) and uninoculated piles (2A and 2B) during composting. Data are means ± SD of two independent experiments.

The evolution of pH during the co-composting is shown in Fig. 2. No significant differences in pH were seen between the inoculated and control piles at any time. A rapid increase in pH was seen in the first 7 days, when values near 8.5 were reached. Thereafter, a slight progressive increase was seen, and pH values near 9.0 were reached after 28 days of composting and were maintained until the end of the monitoring period. This trend agrees with that reported by other authors exploring the co-composting of WOP with different agricultural wastes^16,31,32^. It was previously elucidated that the pH increase, typically occurring between the 18^th^ and 40^th^ days of composting, is due mainly to the release of NH_4_ and partly to CO_2_ elimination due to aeration^33^. This pH trend, which is typically observed during composting of organic substrates, especially of WOP^34,35^, is attributable to the carboxylation of organic anions during aerobic degradation of the organic matter according to some authors^36,37^.

### Evolution of phytotoxicity and water-soluble phenols during co-composting

**Figure 3:**
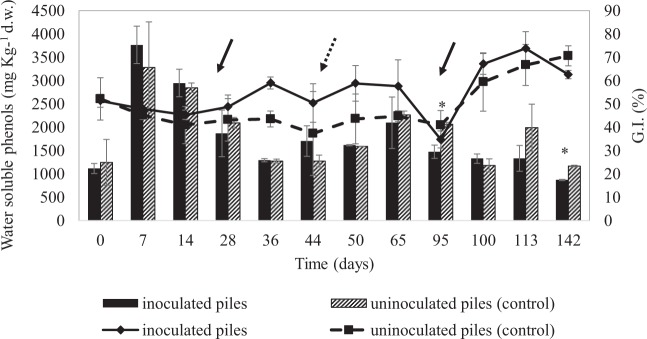
Germination index (GI) (lines) and water soluble phenols content (histograms) of inoculated (1A and 1B) and uninoculated piles (2A and 2B) during composting. Data are means ± SD of two independent experiments. Full shape arrows indicate the time of azotobacter inoculation (after 25 and 94 days of composting); dashed arrow indicates the inoculation of *P. chrysosporium* spores (day 44). *Statistically significant differences (Duncan tests; P < 0.05) between inoculated and uninoculated (control) piles within each sampling time; d.w., dry weight.

The evolution of phytotoxicity and water-soluble phenols during co-composting is depicted in Fig. 3. The germination index (% G.I.) is typically used to evaluate the phytotoxicity of composts and, hence, to assess their maturity. Indeed, high amounts of ammonia, as well as some organic acids and water-soluble compounds, generally occur in immature composts and can hinder the germination of *L. sativum* seeds and their root development. The mean % G.I. of WOP confirmed the high phytotoxicity of this agricultural waste, whose direct application to the soil is not recommended^38^. Regarding the compost material, after 142 days, no significant differences in phytotoxicity were seen between the inoculated and control piles, which reached G.I. values near 70%, starting from initial values slightly above 50% (Fig. 3). According to Echeverria *et al*.^15^, a sample is phytotoxic when its G.I. is lower than 75% at an extract concentration of 30%, whereas for Lasaridi^39^, a sample is phytotoxic when its G.I. is lower than 80% at an extract concentration of 10%. Based on these limits, after 142 days, the compost material of both the inoculated and control piles was still phytotoxic, though G.I. values >60% at an extract concentration of 30% are allowed by the Italian law for agricultural compost application^40^. As far as the scientific literature is concerned, G.I. values either below or above the 80% limit have previously been reported by other authors^4^, suggesting high variability for this parameter depending on numerous factors, such as the initial G.I. value of the raw materials and the duration of composting.

Soluble phenol content is a compost quality indicator as well and is particularly useful for improving and understanding the entire compositing process and for evaluating the evolved level of organic matter^41^. Thanks to their simple structure and small molecular size, water-soluble phenols are more sensitive to the transformations occurring during composting than are the polyphenols generated during the partial degradation of lignin.

When comparing day 0 and day 142, no significant reduction in water-soluble phenol content was seen in the control piles, whereas the final and initial water-soluble phenol content differed significantly in the inoculated piles.

This result seemed to suggest that the inoculation of azotobacteria (at day 25 and 94) and *P. chrysosporium* (at day 44) might have affected the phenol metabolism, and hence the humification process, by enhancing the decomposition of lignocellulose compounds and/or polyphenols. A similar hypothesis was recently formulated by Agnolucci *et al*.^16^ to explain the contribution of a multi-strain starter culture, which included *P. chrysosporium*, to the water-soluble phenol content in mature compost from olive mill solid waste.

Invariably, the final water-soluble phenol content in compost piles in this study were less than 4%. This content level falls within the recommended limit established by several authors for the direct application of organic matter to annual crops for sustaining crop yields and building soil fertility^42^.

When the evolution of phytotoxicity and water-soluble phenol content during composting was evaluated (Fig. 3), additional conclusions could be made. Concerning the water-soluble phenol content, no significant differences were seen overall among the four piles, except for t_95_ and t_142_, when a significantly lower concentration of soluble phenols was seen in the inoculated piles compared with the controls. Moreover, in all four piles, a notable increase of soluble phenols was seen during the thermophilic phase (t_7_–t_14_), followed by a progressive decrease until t_36_, a subsequent gradual increase until t_65_, and a final gradual decrease until the end of the monitoring period. A very similar trend has been reported by Castaldi *et al*.^41^ when analyzing the fraction of water-soluble phenols in compost from municipal solid waste. In that study, an increase of water-soluble phenols was seen after the first two weeks of composting, followed by a gradual decrease, which has been ascribed by the authors to the microbial use of these compounds as either an energy source or as precursors for the synthesis of new molecules. In 2012, Gigliotti *et al*.^43^ reported that during the maturation phase, phenols tend to increase because of the concentration of these compounds.

More recently, an increase in the content of soluble phenols during composting of olive mill wastes has also been reported by Taccari *et al*.^13^. In that study, again after 15 days of composting, an enrichment of these phytotoxic compounds was seen in the piles inoculated with *P. chrysosporium*. The authors^13^ ascribed this increase in the number of phytotoxic compounds to the previously reported capacity of the inoculated basidiomycetes white-rot fungi to liberate additional water-soluble phenols during lignin degradation^44^ via a combination of extracellular organic acids, mediators and enzymes, including laccase (phenol oxidase)^45^. This latter explanation fits well with the overall PCR-DGGE analysis results of the present study, which revealed stable colonization by *Aspergillus nidulans* and *Penicillium chrysogenum* of all four piles from the very early phase until the end of the composting trial (Table 1, Supplementary Fig. 1). Both species are imperfect fungi known to produce lactase, one of the three major enzymes associated with ligninolysis^46^.

**Table 1:** Fungal dynamics in the two inoculated (1A and 1B) and control piles (2A and 2B) as revealed by PCR-DGGE analysis during the composting process.

Experimental piles	Sampling time (day)	*Ascomycota* sp.	*Aspergillus* sp.	*Aspergillus nidulans*	*Candida boidinii*	*Candida stellimalicola*	*Chaetomium elatum*	*Ilyonectria destructans*	*Penicillium chrysogenum*	*Penicillium* sp. *cremeogriseum*	*Penicillium olsonii*	*Phanerochaete* sp.	*Pichia* sp.	*Saccharomyces cerevisiae*	*Sarocladium kiliense*
1A	t0				●								○	●	
	t7			●	●	●			●						
	t23			●	●			○	●						
	t28			●	●		●		●						
	t36			●	●		●		●						
	t44			●	●		●		●						
	t65	○	○	●	●		●		●	●		○			
	t95	○	○	●	●		●		●	●					
	t113	○		●	●				●	●					
1B	t0				●								○	●	
	t7			●	●	●			●						
	t23			●	●			○	●						
	t28			●	●		●		●						
	t36			●	●		●		●						○
	t44			●	●		●		●						○
	t65	○	○	●	●		●		●	●		○			
	t95	○	○	●	●		●		●	●					
	t113	○		●	●				●	●					
2A	t0		○		●				●		●		○	●	
	t7			●	●	●			●						
	t23			●	●			○	●						
	t28			●	●		●		●						
	t36			●	●		●		●						
	t44			●	●		●		●						
	t65	○	○	●	●		●		●	●					
	t95	○	○	●	●		●		●	●					
	t113	○		●	●				●						
2B	t0				●				●				○	●	
	t7			●	●	●			●						
	t23			●	●				●						
	t28			●	●		●		●						
	t36			●	●		●		●						
	t44			●	●		●		●						○
	t65	○	○	●	●		●		●	●					
	t95	○	○	●	●		●		●	●					
	t113	○		●	●				●						

●DGGE bands resulting from the analysis of the DNA extracted directly from samples showing >97% of similarity with the sequences of the closest relatives found by a BLAST search in the GenBank database.

○ DGGE bands resulting from the analysis of the DNA extracted directly from samples showing ≤97% of similarity with the sequences of the closest relatives found by a BLAST search in the GenBank database.

1A and 1B: experimental piles inoculated with *Azotobacter chroococcum* and *Phanerochaete chrysosporium*; 2A and 2B: control piles.

Besides to the latter enzymes, fungi are also known to produce polyphenoloxidases during either the thermophilic phase^47^ or, according to other authors, during the initial activation stage or after the thermophilic stage^48^.

Regarding the phytotoxicity evolution of the compost piles, a negative trend with water-soluble phenols was seen (Fig. 3). This result is mainly ascribable to the previously reported higher toxicity of simple water-soluble phenols as compared with more complex polymerized compounds^13^.

### Microbiological analyses

**Figure 4:**
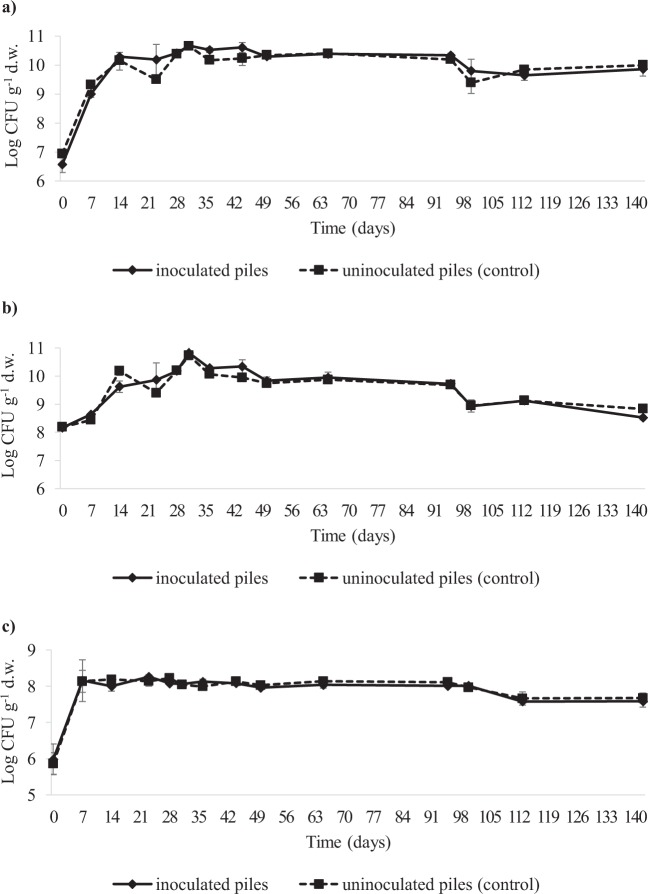
Viable counts of total mesophilic aerobes (panel a), total thermophilic aerobes (panel b) and eumycets (panel c) assessed at different time points (at 0, 7, 14, 23, 28, 31, 36, 44, 50, 65, 95, 100, 113 and 142 days) during the composting process. Inoculated piles (1A and 1B) underwent inoculation with azotobacteria and spores of *Phanerochaete chrysosporium*, whereas uninoculated piles (2A and 2B) were used as controls. Error bars indicate ± SD of two independent experiments.

Microbiological analysis of WOP revealed high loads of total mesophilic aerobes and total eumycetes attesting at 7.36 ± 0.04 and 6.59 ± 0.10 Log CFU g^−1^, respectively. A high level of bacterial and yeast colonization in the same type of substrate was previously reported by Echeverria *et al*.^15^. The results of viable cell counting in the samples of compost collected during composting are shown in Fig. 4. As far as the inoculated and control piles are concerned, as a general trend, robust microbial colonization was seen during composting, irrespective of inoculation with the selected starter strains. Moreover, comparable loads of mesophilic and thermophilic bacteria, as well as of eumycetes, were observed during composting of both the microbiologically enriched and control piles. In more detail, a rapid increase in the viable counts of the three microbial groups was seen during the first 7 days, followed by stabilization after 14 days, when the concentrations of mesophilic aerobes, thermophilic aerobes and eumycetes plateaued near 10.0, 8.5 and 7.5 Log CFU g^−1^, respectively. Except for the thermophilic aerobes, which showed a progressive decrease from day 100 to the end of the composting trial, when loads approximately 8.5 Log CFU g^−1^ were reached, loads of mesophilic aerobes and eumycetes remained almost stable from day 14 until the end of the composting trial. In accordance to what was previously suggested by Principi *et al*.^49^ about the limited significance of viable cell counting of microbes in compost material, no correlation was seen between the internal pile temperature and the amount of cultivable microbial biomass during co-composting of WOP with straw and wood chips as bulking agents. The same result was previously observed by Echeverria *et al*.^15^ when enumerating total bacteria, actinobacteria, yeasts and microfungi in microbially enhanced compost from wet olive husks.

The viable count data were largely in accordance with the denaturing gradient gel electrophoresis (DGGE) profiles. Indeed, an equally similar trend in the evolution of the microbiota in the four piles was revealed by PCR-DGGE and sequencing of DNAs eluted from selected bands (Supplementary Figs 1 and 2; Tables 1 and 2). As far as the bacterial populations are concerned, a stable microbial succession was seen in the four piles, with a few species that occurred in the initial matrix, namely, *Acetobacter nitrogenifigens, Acinetobacter radioresistens, Acinetobacter variabilis, Gluconobacter albidus, Reyranella soli* and *Swingsia samuiensis*, either co-existing or being replaced by other species, namely, *Acinetobacter gandensis*, *Paracoccus laeviglucosivorans*, during the thermophilic phase. In the final maturation phase, the latter microorganisms were replaced by additional species, namely *Pseudomonas guguanensis, Blastochloris viridis, Ferrovibrio denitrificans, Luteimonas terricola, Nitrosomonas communis, Novispirillum itersonii, Paracoccus kocurii, Pseudorhodoplanes sinuspersici, Pseudoxanthomonas suwonensis, Qingshengfania soli, R. soli*, and *Rhizobium endolithicum*. A few species among those dominating during maturation were detected at well-defined time points, namely, *B. viridis*, *F. denitrificans* and *P. kocurii*, which were identified at t_44_, t_65_ to t_95_, and t_65_ to t_113_, respectively. By contrast, other species were identified either throughout the whole maturation phase, such as *Pseudoxanthomonas suwonensis*, detected from t_7_ (pile 1B) or t_23_ (piles 1A, 2A and 2B) until the end of the monitoring period, or during the late maturation phase, such as *L. terricola*, *N. communis*, *N. itersonii*, *P. kocurii*, and *Q. soli*.

**Table 2:** Bacterial dynamics in the two inoculated (1A and 1B) and control piles (2A and 2B) as revealed by PCR-DGGE analysis during the composting process.

Experimental pile	Sampling time (day)	*Acetobacter nitrogenifigens*	Acetohalobium arabaticum	Acinetobacter gandensis	Acinetobacter radioresistens	Acinetobacter variabilis	Actimicrobium antarcticum	*Blastochloris viridis*	*Ferrovibrio denitrificans*	*Gluconobacter albidus*	*Luteimonas terricola*	*Nitrosomonas communis*	*Novispirillum itersonii*	*Novosphingobium acidiphilum*	*Paracoccus kocurii*	*Paracoccus laeviglucosivorans*	*Pseudomonas guguanensis*	*Pseudorhodoplanes sinuspersici*	*Pseudoxanthomonas suwonensis*	*Qingshengfania soli*	*Reyranella soli*	*Rhizobium endolithicum*	*Rhodoligotrophos jinshengii*	*Rhodothalassium salexigens*	*Swingsia samuiensis*
1A	t0	●			●	●				●											●				●
	t7			○												●					●				●
	t23		●				○									●			●						●
	t28																		●						
	t36																○		●						
	t44							○									○		●						
	t65								○		●	●	●		○			○	●	●					
	t95								○		●	●	●		○			○	●	●					
	t113										●	●	●		○				●	●	●	●			
1B	t0	●			●	●				●											●				●
	t7			○												●					●				●
	t23		●				○									●			●						
	t28																○		●						
	t36																		●						
	t44							○											●						
	t65								○		●	●	●		○			○	●	●					
	t95								○		●	●	●		○			○	●	●					
	t113										●	●	●		○				●	●	●	●			
2A	t0	●			●	●				●				○							●		○		●
	t7			○												●								○	●
	t23		●				○									●			●						
	t28																		●						
	t36																○		●						
	t44							○											●						
	t65								○		●	●	●		○			○	●	●					
	t95								○		●	●	●		○			○	●	●					
	t113										●	●	●		○				●	●	●	●			
2B	t0	●			●	●				●				○							●		○		●
	t7		●	○			○									●			●		●				●
	t23		●				○									●			●						
	t28																○		●						
	t36																		●						
	t44							○											●						
	t65								○		●	●	●		○			○	●	●					
	t95								○		●	●	●		○			○	●	●					
	t113										●	●	●		○				●	●	●	●			

●DGGE bands resulting from the analysis of the DNA extracted directly from samples and showing >97% of similarity with the sequences of the closest relatives found by a BLAST search in the GenBank database.

○DGGE bands resulting from the analysis of the DNA extracted directly from samples and showing ˂ 97% of similarity with the sequences of the closest relatives found by a BLAST search in the GenBank database

1A and 1B: experimental piles inoculated with azotobacteria and spores of *Phanerochaete chrysosporium*; 2A and 2B: control piles.

Regarding eumycetes, a quite stable evolution of the population was again seen in the four piles. In more detail, some species were detected during the whole composting process, such as *Aspergillus nidulans, Candida boidinii* and *P. chrysogenum*. Other species apparently occurred only in the initial matrices (*Pichia* sp., *Saccharomyces cerevisiae*), the thermophilic phase (*Candida stellimalicola*) or at defined time points during the maturation phase (*Ascomycota* sp., *Aspergillus* sp., and *Penicillium cremeogriseum*, detected from t_65_ or t_95_ until t_113_, and *Ilyonectria destructans*, detected only at t_65_).

Interestingly, neither *Azotobacter* nor *Phenerochete* could be detected by sequencing of DNA eluted from the excised DGGE bands. This might be explained by the lack of dominance of the inoculated strains in the piles, which in turn might be feasible due to an initially low load or, alternatively, to lower competitive fitness of the starter strains with respect to the autochthonous microorganisms already residing in the matrices to be composted.

The lack of DGGE bands ascribable to the inoculated species seems to support what other authors have previously suggested about the still debated use of starters to speed up and guide the composting process^50–52^. Among those supporting the infectiveness of starter cultures for composting, Golueke^53^ suggested that inoculants introduced into compost mixtures are already colonized by diverse communities of organisms, each of which rapidly multiplies under the most favorable conditions. Hence, mechanisms such as substrate enzymatic induction and natural selection rapidly lead to the dominance of endemic specialized populations.

When compared to the results collected in other published studies relying on culture-independent approaches, the data collected in the present study confirmed the high variation in the microbial diversity characterizing the different phases of the composting process, as well as the progressive stabilization of the microbial population as the compost piles reach maturity^16^.

Though it is difficult to determine whether the species identified by PCR-DGGE analysis were metabolically active, as extracted genomic DNA could be from resting cells within the samples, the dynamic temporal shifts of DGGE bands suggest that DNA turnover necessarily occurred during the composting. This, in turn, indicates that the microbial species identified through sequencing of the selected DGGE bands were actively growing. Indeed, it is conceivable that if sequenced DGGE bands belonged to vegetative cells, those visualized at the first time points should have been detected throughout the monitoring period. Since this was not the case, the initial hypothesis must be rejected. The same conclusion can be reasonably drawn for eumycetes.

Despite the observed microbial succession, the causative factors of the shift in microbial community composition remain unclear, and changes in substrate availability, temperature, or pH might have influenced these community changes.

The bacterial species identified in the present study included microorganisms with very heterogeneous metabolic activities and that originated from various sources. These include obligate aerobes (*A. variabilis, A. gandensis*), acetic acid bacteria (*G. albidus, S. samuiensis*), bacteria involved in the nitrogen cycle (A. *nitrogenifigens, N. communis, P. kocurii, Q. soli)*, human and animal commensal bacteria (*A. radioresistens, A. variabilis, A. gandensis*) and, as expected, bacteria typically associated with soil environments (*R. soli, P. laeviglucosivorans, L. terricola, Q. soli*) and even composted agricultural wastes (*P. suwonensis*). Except for *A. radioresistens*, all these bacteria are novel species, mostly identified in the last few years and, hence, not yet fully characterized, thus rendering the comprehension of their contribution in the composting process particularly challenging.

As far as the fungal communities are concerned, again, a very similar evolution was seen in the four composts, with no differences among the enriched and control piles. Overall, a relatively low fungal species richness was seen at any given monitoring time; however, the community structure of eumycetes was dynamic and paralleled changes in the bacterial community structure^54^.

Eumycetes play a key role in compost, being prevalent in this ecosystem, thanks to their strong ability to degrade lignocellulose compounds, which are particularly abundant in agricultural wastes. A few genera are dominant in this compost, namely, *Aspergillus*, *Mucor*, *Penicillium*, *Humicola*, *Caetomium* and *Thermomyces*. Overall, the dominant species identified in this study have previously been detected in compost. *A. nidulans* and *P. chrysogenum* have been isolated from spent mushroom compost^45^. Selected cultures of the latter species, isolated from different composting processes, were found to tolerate heavy metals in multipolluted aqueous systems, as well as to remove these metals with high detoxification ratios^50^. Analogously, isolates of *C. boidinii* collected from two-phase decanter system olive oil waste were proven to degrade toxic substances, such as phenols, in a fed-batch microcosm system^55^. More intriguingly, both *A. nidulans* and *P. chrysogenum* are known to produce laccase^46^, a key enzyme in the ligninolytic process, thus suggesting a key role for these microorganisms in the transformations of organic matter during the composting of WOP.

## Conclusions

To date, the composting of WOP has not been thoroughly elucidated, and most aspects regarding microbial population dynamics remain largely unknown. In this study, new information was attained by exploring the effects of azotobacteria and *P. chrysosporium* inoculation on the composting of piles added with WOP. Overall, only a modest impact of the starter strains on the physical, chemical and microbiological properties of the mature composts was seen, except for a significantly lower content of water-soluble phenols in the inoculated piles after 142 days. However, an intense evolution of these compounds was seen in all piles, thus suggesting an active role of the heterogeneous resident microbiota.

## Supplementary information

Supplementary Figure 1

Supplementary Figure 2

## References

[1] Otles S, Selek I (2012). Treatment of olive mill wastewater and the use of polyphenols obtained after treatment. Int. J. Food Stud..

[2] Gómez-Muñoz, B., Hatch, D. J., Bol, R. & García-Ruiz, R. The compost of olive mill pomace: from a waste to a resource - environmental benefits of its application in olive oil groves, in Sustainable Development – Authoritative and Leading Edge Content for Environmental Management, ed Sime Curkovic (InTech), 459–484 (2012).

[3] Altieri R, Esposito A (2010). Evaluation of the fertilizing effect of olive mill waste compost in short-term crops. Int. Biodeter. Biodegr..

[4] Michailides M, Panagopoulos P, Akratos CS, Tekerlekopoulou AG, Vayenas DV (2011). A full-scale system for aerobic biological treatment of olive mill wastewater. J. Chem. Technol. Biotechnol..

[5] Tuomela M, Vikman M, Hatakka A, Itavaara M (2000). Biodegradation of lignin in a compost environment: a review. Bioresour. Technol..

[6] Garcia-Barrionuevo A, Moreno E, Quevedo-Sarmiento J, Gonzalez-Lopez J, Ramos-Cormenzana A (1992). Effect of wastewaters from olive oil mills (alpechin) on Azotobacter nitrogen fixation in soil. Soil Biol. Biochem..

[7] Balis C, Chatzipavlidis J, Flouri F (1996). Olive mill waste as a substrate for nitrogen fixation. Int. Biodeterior. Biodegrad..

[8] Papadelli (1996). Biochemical and molecular characterization of an *Azobacter vinelandii* strain with respect to its ability to grow and fix nitrogen in olive mill wastewater. Int. Biodeterior. Biodegrad..

[9] Piperidou C (2000). Bioremediation of olive mill wastewater: chemical alterations induced by *Azobacter vinelandii*. J. Agric. Food Chem..

[10] Fiorelli F, Pasetti L, Galli E (1996). Fertility-promoting metabolites produced by Azotobacter vinelandii grown on olive-mill wastewaters. Int. Biodeterior. Biodegrad..

[11] Niaounakis, M. & Halvadakis, C. P. Olive processing waste management: Literature review and patent survey, second ed. Elsevier, Amsterdam, The Netherlands, pp 200–202 (2006).

[12] Ahmadi M, Vahabzadeh F, Bonakdarpour B, Mehranian M, Mofarrah E (2006). Phenolic removal in olive oil mill wastewater using loofah-immobilized Phanerochaete chrysosporium. World J. Microbiol. Biotechnol..

[13] Taccari M, Stringini M, Comitini F, Ciani M (2009). Effect of *Phanerochaete chrysosporium* inoculation during maturation of co-composted agricultural wastes mixed with olive mill wastewater. Waste Manag..

[14] Haddadin MSY, Haddadin J, Arabiyat OI, Hattar B (2009). Biological conversion of olive pomace into compost by using Trichoderma harzianum and Phanerochaete chrysosporium. Bioresour. Technol..

[15] Echeverria (2012). Microbial-enhanced composting of wet olive husks. Bioresour. Technol..

[16] Agnolucci (2013). Microbially-enhanced composting of olive mill solid waste (wet husk): Bacterial and fungal community dynamics at industrial pilot and farm level. Bioresour. Technol..

[17] Aquilanti (2014). Integrated biological approaches for olive mill wastewater treatment and agricultural exploitation. Int. Biodet. Biodegrad..

[18] Zucconi F, Pera A, Forte M, De Bertoldi M (1981). Evaluating toxicity of immature compost. Biocycle..

[19] Mari I, Ehaliotis C, Kotsou M, Balis C, Georgakakis D (2003). Respiration profiles in monitoring the composting of by-products from the olive oil agro-industry. Biores. Technol..

[20] Garofalo (2015). Bacteria and yeast microbiota in milk kefir grains from different Italian regions. Food Microbiol..

[21] Makimura (1999). Phylogenetic classification and species identification of dermatophyte strains based on DNA sequences of nuclear ribosomal internal transcribed spacer 1 regions. J. Clin. Microbiol..

[22] Alessandria (2010). Microbiota of the Planalto de Bolona: an artisanal cheese produced in uncommon environmental conditions in the Cape Verde Islands. World J. Microbiol. Biotechnol..

[23] Cocolin L, Bisson LF, Mills DA (2000). Direct profiling of the yeast dynamics in wine fermentations. FEMS Microbiol. Lett..

[24] Ampe F, Ben Omar N, Moizan C, Wacher C, Guyot JP (1999). Polyphasic study of the spatial distribution of microorganisms in Mexican pozol, a fermented maize dough, demonstrates the need for cultivation-independent methods to investigate traditional fermentations. Appl. Environ. Microbiol..

[25] Sheffield VC, Cox DR, Lerman LS, Myers RM (1989). Attachment of a 40-base pairs G þ C rich sequence (GC clamp) to genomic DNA fragments by the polymerase chain reaction results in improved detection of single-base changes. Protoc. Natl. Acad. Sci. USA.

[26] Osimani A, Garofalo C, Aquilanti L, Milanović V, Clementi F (2015). Unpasteurised commercial boza as a source of microbial diversity. Int. J. Food Microbiol..

[27] Taccari (2016). Microbial diversity of type I sourdoughs prepared and back-slopped with wholemeal and refined soft (*Triticum aestivum*) wheat flours. J. Food Sci..

[28] Garofalo (2017). Study of the bacterial diversity of foods: PCR-DGGE versus LH-PCR. Int. J. Food Microbiol..

[29] Alburquerque JA, Gonzàlves J, Garcıa D, Cegarra J (2004). Agrochemical characterisation of “alperujo”, a solid byproduct of the two-phase centrifugation method for olive oil extraction. Bioresour. Technol..

[30] Leouifoudi I (2014). Identification and characterisation of phenolic compounds extracted from Moroccan olive mill wastewater. Food Sci. Technol. (Campinas)..

[31] Alfano G, Belli C, Lustrato G, Ranalli G (2008). Pile composting of two phase centrifuged olive husks residues: technical solutions and quality of cured compost. Bioresour. Technol..

[32] Cayuela ML, Sanchez-Monedero MA, Roig A (2010). Two-phase olive mill waste composting: enhancement of the composting rate and compost quality by grape stalks addition. Biodegradation..

[33] Ekinci K, Keener HM, Elwell DL (2000). Composting short paper fiber with broiler litter and additives. Part I: effects of initial pH and carbon/nitrogen ratio on ammonia emission. Compost Sci. Util..

[34] Cayuela ML, Bernal MP, Roig A (2004). Composting olive mill waste and sheep manure for orchard use. Compost. Sci. Util..

[35] Alburquerque JA, Gonzálvez J, García D, Cegarra J (2006). Measuring detoxification and maturity in compost made from “alperujo”, the solid by-product of extracting olive oil by the two-phase centrifugation system. Chemosphere..

[36] Cayuela ML, Millner PD, Meyer SLF, Roig A (2008). Potential of olive mill wastes as biobased pesticides against weeds, fungi and nematodes. Sci. Total Environ..

[37] Cayuela ML, Mondini C, Sánchez-Monedero MA, Roig A (2008). Chemical properties and hydrolytic enzyme activities for the characterization of two-phase olive mill waste composting. Bioresour. Technol..

[38] Echeverria (2011). Composting wet olive husks with a starter based on oil-depleted husks enhances compost humification. Compost Sci. Util..

[39] Lasaridi K, Loanna P, Kotsou M, Georg P, Thakis M (2006). Quality assessment of compost in the Greek market: the need for standards and quality assurance. J. Environ. Manage..

[40] Legislative Decree 75 of 29th April “Revision of the Discipline on Fertilizers”, available online on, https://www.politicheagricole.it (2010).

[41] Castaldi P, Garau G, Melis P (2008). Maturity assessment of compost from municipal solid waste through the study of enzyme activities and water-soluble fractions. Waste Manag..

[42] Palm CA, Gachengo CN, Delve RJ, Cadisch G, Giller KE (2001). Organic inputs for soil fertility management in tropical agroecosystems: application of an organic resource database. Agric. Ecosyst. Environ..

[43] Gigliotti (2012). Co-composting of olive husks with high moisture contents: Organic matter dynamics and compost quality. Int. Biodeterior. Biodegradation..

[44] Kapich AN (2004). Effect of lignocellulose-containing substrates on production of ligninolytic peroxidases in submerged cultures of Phanerochaete chrysosporiumME-446. Enzyme Microb. Technol..

[45] Dashtban M, Schraft H, Syed TA, Qin W (2010). Fungal biodegradation and enzymatic modification of lignin. Int. J. Biochem. Mol. Biol..

[46] Arora DS, Sharma RK (2010). Ligninolytic fungal laccases and their biotechnological applications. Appl. Biochem. Biotechnol..

[47] Madejón E, Galli E, Tomati U (1998). Composting of wastes produced by low water consuming olive mill technology. Agrochimica..

[48] Mayende L, Wilhelmi B, Pletschke B (2006). Cellulases (CMCases) and polyphenol oxidases from thermophilic. Soil Biol. Biochem..

[49] Principi P (2003). Microbiological aspects of humid husk composting. J. Environ. Sci. Health..

[50] Vargas-García Mdel C, López MJ, Suárez-Estrella F, Moreno J (2012). Compost as a source of microbial isolates for the bioremediation of heavy metals: *in vitro* selection. Sci. Total Environ..

[51] Acevedo, M., Acevedo, L., Restrepo-Sánchez, N. & Peláez, C. The inoculation of microorganisms in composting processes: need or commercial strategy? *Livestock Research for Rural Development* **17**(12) (2005).

[52] Fan YV, Klemeš JJ, Lee CT, Ho CS (2018). Efficiency of microbial inoculation for a cleaner composting technology. Clean Technol. Environ..

[53] Golueke, C. G. Composting. A Study of the Process and its Principles. Rodale Press, Emmaus, USA (1972).

[54] Kleyn JG, Wetzler TF (1981). The microbiology of spent mushroom compost and its dust. Can. J. Microbiol..

[55] Giannoutsou EP, Meintanis C, Karagouni AD (2004). Identification of yeast strains isolated from a two-phase decanter system olive oil waste and investigation of their ability for its fermentation. Bioresour. Technol..

