# Supplementary material for: Effect of inoculated azotobacteria and Phanerochaete chrysosporium on the composting of olive pomace: Microbial community dynamics and phenols evolution
Source: Sci Rep. 2019 Nov 18;9:16966. doi: 10.1038/s41598-019-53313-z (PMC6861245; doi:10.1038/s41598-019-53313-z)

Effect of inoculated azotobacteria and *Phanerochaete chrysosporium* on the composting of olive pomace: Microbial community dynamics and phenols evolution

Vesna Milanović[1], Andrea Osimani[1], Federica Cardinali[1], Manuela Taccari[1], Cristiana Garofalo[1], Francesca Clementi[1], Selim Ashoor[2,3], Massimo Mozzon[1], Roberta Foligni[1], Laura Canonico[2], Maurizio Ciani[2], Lucia Aquilanti[1,*]

[1] Dipartimento di Scienze Agrarie, Alimentari ed Ambientali, Università Politecnica delle Marche, 60131 Ancona, Italy
[2] Dipartimento Scienze della Vita e dell'Ambiente, Università Politecnica delle Marche, Via Brecce Bianche, 60131 Ancona, Italy.
[3] Department of Agricultural Microbiology, Faculty of Agriculture, Ain Shams University, Cairo, Egypt

Corresponding author:
Prof. Lucia Aquilanti, PhD
e-mail: l.aquilanti@univpm.it
Dipartimento di Scienze Agrarie, Alimentari ed Ambientali, Università Politecnica delle Marche, 60131 Ancona, Italy

Supplementary Figure 2. DGGE profiles of amplicons produced with primers 338fGC-518r from the bacterial DNAs extracted from the two experimental piles inoculated with azotobacteria and spores of *Phanerochaete chrysosporium* (1A and 1B) and the two control piles (2A and 2B) at different time points during composting process. Panel a) sampling time points T0 (0 days), T7 (7 days) and T23 (23 days); panel b) sampling time points T28 (28 days), T36 (36 days) and T44 (44 days); panel c) sampling time points T65 (65 days), T95 (days) and T113 (113 days). The bands indicated by the letters were excised, re-amplified and subjected to sequencing. Ladder (L) refers to the mixture of three *Azotobacter chrooococcum* strains (208, 220, 225).

a)

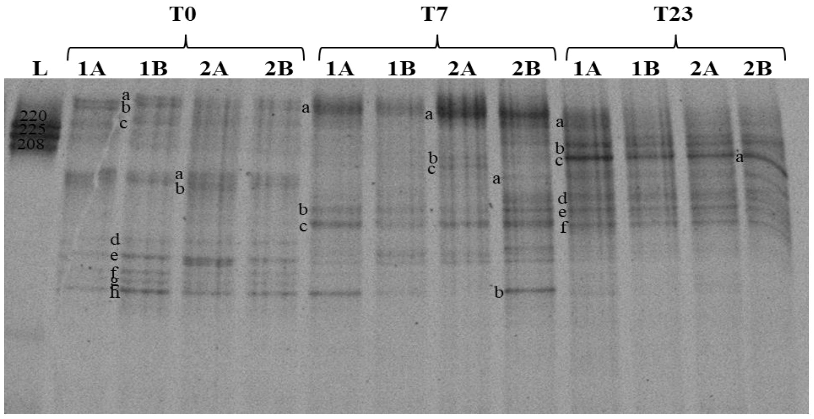

b)

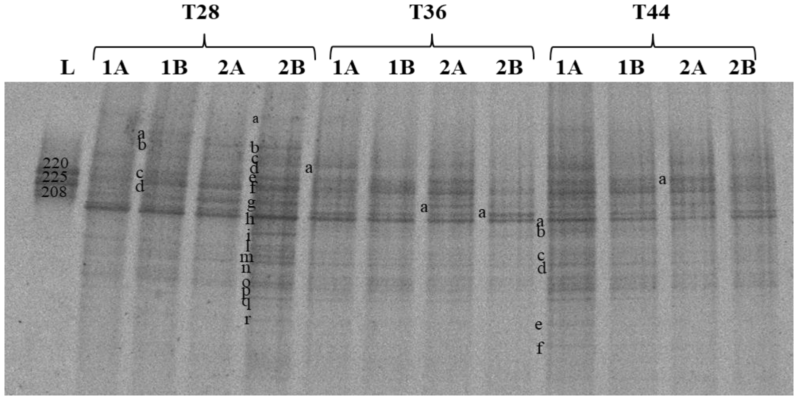

c)

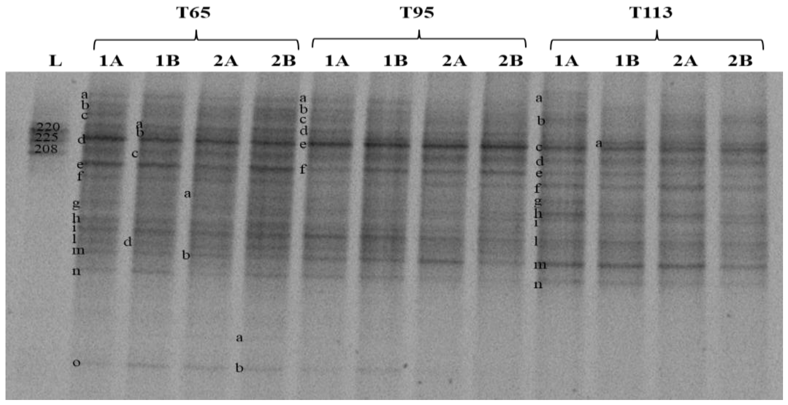

Supplement: Supplementary file 2 — Supplementary Figure 2 [file 41598_2019_53313_MOESM2_ESM.pdf]
